# (2*E*)-2-{[3-Methyl-5-(2-naphth­yloxy)-1-phenyl-1*H*-pyrazol-4-yl]methyl­idene}hydrazinecarbothio­amide monohydrate

**DOI:** 10.1107/S1600536812039815

**Published:** 2012-09-29

**Authors:** Hoong-Kun Fun, Tze Shyang Chia, Shobhitha Shetty, Balakrishna Kalluraya, Nithinchandra

**Affiliations:** a X-ray Crystallography Unit, School of Physics, Universiti Sains Malaysia, 11800 USM, Penang, Malaysia; b Department of Pharmaceutical Chemistry, College of Pharmacy, King Saud University, Riyadh 11451, Saudi Arabia; c Department of Studies in Chemistry, Mangalore University, Mangalagangotri, Mangalore 574 199, India

## Abstract

In the title compound, C_22_H_19_N_5_OS·H_2_O, the naphthalene ring system and the benzene ring [dihedral angle = 85.19 (8)°] make dihedral angles of 87.02 (9) and 14.41 (10)°, respectively, with the pyrazole ring. The mean plane through the 2-methyl­enehydrazinecarbothio­amide group [C—N—N—C(=S)—N; maximum deviation = 0.022 (1) Å] is slightly twisted from the pyrazole ring [dihedral angle = 5.60 (11)°]. In the crystal, mol­ecules are linked by N—H⋯S, N—H⋯O, O—H⋯S, O—H⋯N and C—H⋯S hydrogen bonds into sheets parallel to the *ab* plane. π–π inter­actions are also observed [centroid-to-centroid distances = 3.7778 (12) and 3.7010 (12) Å].

## Related literature

For the biological activities of pyrazoles and their derivatives, see: Rai *et al.* (2008 ▶); Hall *et al.* (2008 ▶); Isloor *et al.* (2009 ▶); Girisha *et al.* (2010 ▶). For the stability of the temperature controller used for data collection, see: Cosier & Glazer (1986 ▶).
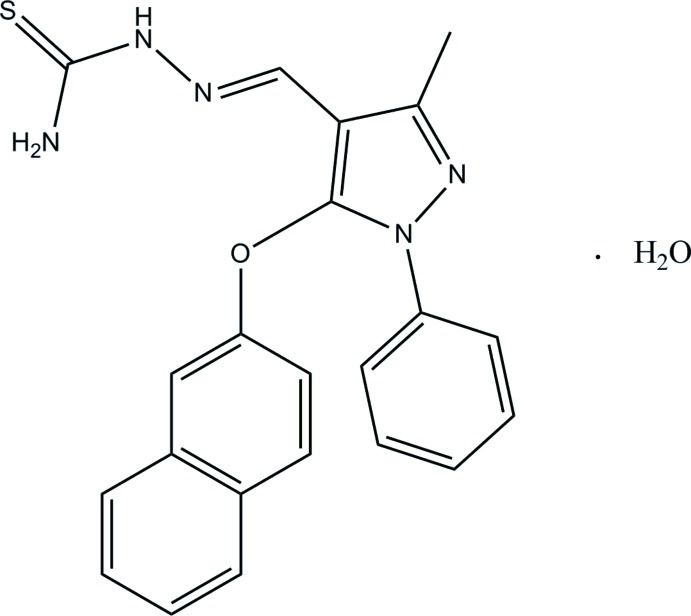


## Experimental

### 

#### Crystal data

C_22_H_19_N_5_OS·H_2_O
*M* *_r_* = 419.50
Triclinic, 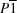

*a* = 7.9384 (2) Å
*b* = 11.1512 (2) Å
*c* = 13.0325 (3) Å
α = 113.481 (1)°
β = 90.942 (1)°
γ = 107.359 (1)°
*V* = 997.81 (4) Å^3^
*Z* = 2
Mo *K*α radiation
μ = 0.19 mm^−1^
*T* = 100 K
0.41 × 0.22 × 0.17 mm

#### Data collection

Bruker SMART APEXII CCD diffractometer
Absorption correction: multi-scan (*SADABS*; Bruker, 2009 ▶) *T*_min_ = 0.925, *T*_max_ = 0.967
15860 measured reflections
4542 independent reflections
3499 reflections with *I* > 2σ(*I*)
*R*_int_ = 0.031

#### Refinement

*R*[*F* ^2^ > 2σ(*F* ^2^)] = 0.044
*wR*(*F* ^2^) = 0.124
*S* = 1.04
4542 reflections
292 parameters
H atoms treated by a mixture of independent and constrained refinement
Δρ_max_ = 0.33 e Å^−3^
Δρ_min_ = −0.33 e Å^−3^

### 

Data collection: *APEX2* (Bruker, 2009 ▶); cell refinement: *SAINT* (Bruker, 2009 ▶); data reduction: *SAINT*; program(s) used to solve structure: *SHELXTL* (Sheldrick, 2008 ▶); program(s) used to refine structure: *SHELXTL*; molecular graphics: *SHELXTL*; software used to prepare material for publication: *SHELXTL* and *PLATON* (Spek, 2009 ▶).

## Supplementary Material

Crystal structure: contains datablock(s) global, I. DOI: 10.1107/S1600536812039815/rz5007sup1.cif

Structure factors: contains datablock(s) I. DOI: 10.1107/S1600536812039815/rz5007Isup2.hkl

Supplementary material file. DOI: 10.1107/S1600536812039815/rz5007Isup3.cml

Additional supplementary materials:  crystallographic information; 3D view; checkCIF report

## Figures and Tables

**Table 1:** Hydrogen-bond geometry (Å, °)

*D*—H⋯*A*	*D*—H	H⋯*A*	*D*⋯*A*	*D*—H⋯*A*
N4—H1N4⋯S1^i^	0.93 (3)	2.56 (3)	3.466 (2)	165 (3)
N5—H2N5⋯O1*W*	0.89 (3)	1.94 (3)	2.805 (3)	164 (3)
O1*W*—H2*W*1⋯S1^ii^	0.94 (4)	2.58 (4)	3.397 (2)	145 (3)
O1*W*—H1*W*1⋯N2^iii^	0.89 (4)	2.01 (4)	2.876 (3)	167 (4)
C20—H20*A*⋯S1^i^	0.95	2.87	3.741 (2)	153

Symmetry codes: (i) 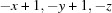; (ii) 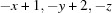; (iii) 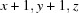.

**Fig. 1.**
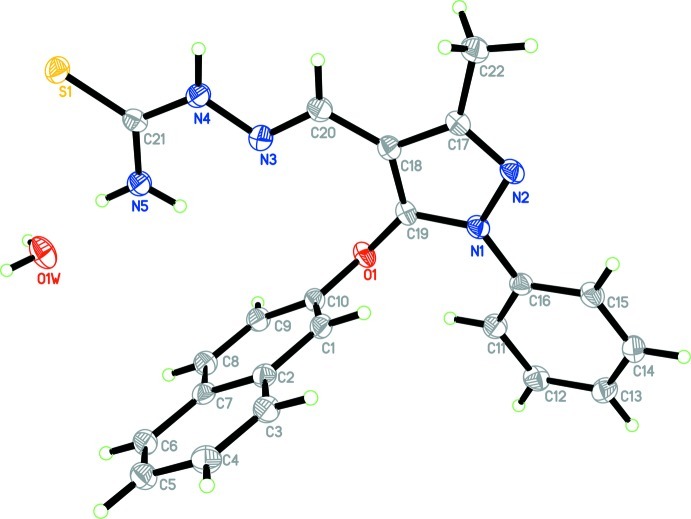
The molecular structure of the title compound with 50% probability displacement ellipsoids.

**Fig. 2.**
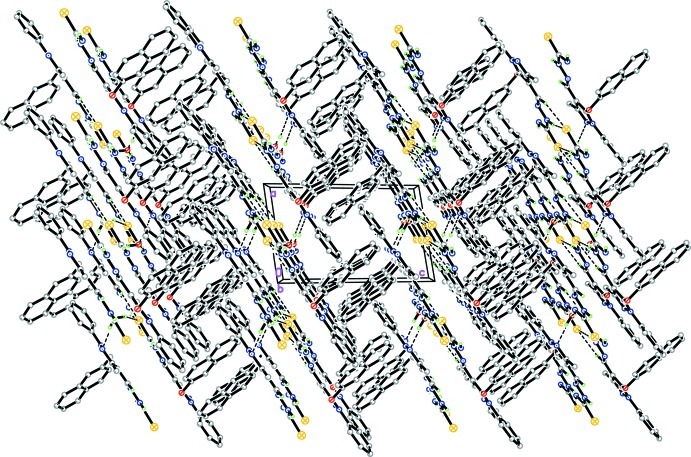
The crystal packing of the title compound. The dashed lines represent the hydrogen bonds. H atoms not involved in hydrogen bonding have been omitted.

## supplementary crystallographic information

### Comment

Pyrazoles and their derivatives play an important role in medicinal chemistry (Rai *et al.*, 2008). Several derivatives of pyrazole are of pharmaceutical interest due to their analgesic action. Pyrazole molecules also exhibit anticancer (Hall *et al.*, 2008), anti-inflammatory, antidepressant, anticonvulsant and anti-HIV properties (Isloor *et al.*, 2009). During the past years, considerable evidence has been accumulated to demonstrate the efficacy of pyrazole derivatives. The incorporation of an aryl system into the pyrazole ring enhances the biological activities to a great extent (Girisha *et al.*, 2010). In view of the importance of these molecules, we report herein the crystal structure of the title compound.

The asymmetric unit of the title compound (Fig. 1) consists of one (2*E*)-2-{[3-methyl-5-(2-naphthayloxy)-1-phenyl-1*H*-pyrazol-4-yl]methylidene}hydrazinocarbothioamide molecule and one water molecule. The C1–C10 naphthalene ring system [maximum deviation = 0.025 (2) Å at atom C10] and C11–C16 benzene ring [dihedral angle between them = 85.19 (8)°] make dihedral angles of 87.02 (9)° and 14.41 (10)°, respectively, with the N1/N2/C17–C19 pyrazole ring. The 2-methylenehydrazinecarbothioamide group [C20/N3/N4/C21/S1/N5; maximum deviation = 0.022 (1) Å at atom N4] is slightly twisted from pyrazole ring as indicated by the dihedral angle of 5.60 (11)°. In the crystal (Fig. 2), molecules are linked by intermolecular N4—H1N4···S1, N5—H2N5···O1W, O1W—H2W1···S1, O1W—H1W1···N2 and C20—H20A···S1 hydrogen bonds into sheets parallel to (001) plane. π–π interactions are also observed with *Cg*1···*Cg*2 = 3.7778 (12) Å [symmetry code = -*x*, 2 - *y*, 1 - *z*] and *Cg*3···*Cg*3 = 3.7010 (12) Å [symmetry code = -1 - *x*, 1 - *y*, 1 - *z*], where *Cg*1, *Cg*2 and *Cg*3 are the centroids of the C1/C2/C7–C10, C2–C7 and C11–C16 rings, respectively.

### Experimental

The title compound was obtained by refluxing a mixture of 3-methyl-5-(2-naphthyloxy)-1-phenyl-1*H*-pyrazole-4-carbaldehyde (0.01 mol) and thiosemicarbazide (0.01 mol) in ethanol (30 ml) with 3 drops of concentrated sulfuric acid for 1 h. Excess ethanol was removed from the reaction mixture under reduced pressure. The solid product obtained was filtered, washed with ethanol and dried. Yellow block-shaped crystals suitable for X-ray analysis were obtained by slow evaporation from ethanol–*N*,*N*-dimethylformamide (3:1 *v*/*v*) solution.

### Refinement

The N- and O-bound H atoms were located in a difference Fourier map and refined freely [N4—H1N4 = 0.93 (3) Å, N5—H2N5 = 0.89 (3) Å, N5—H1N5 = 0.88 (3) Å, O1W—H2W1 = 0.94 (3) Å, O1W—H1W1 = 0.88 (3) Å]. The remaining H atoms were positioned geometrically [C—H = 0.95 and 0.98 Å] and refined using a riding model with *U*_iso_(H) = 1.2 or 1.5*U*_eq_(C). A rotating group model was applied to the methyl group.

### Crystal data

**Table:**

C_22_H_19_N_5_OS·H_2_O	*Z* = 2
*M**_r_* = 419.50	*F*(000) = 440
Triclinic, *P*1	*D*_x_ = 1.396 Mg m^−^^3^
Hall symbol: -P 1	Mo *K*α radiation, λ = 0.71073 Å
*a* = 7.9384 (2) Å	Cell parameters from 5898 reflections
*b* = 11.1512 (2) Å	θ = 2.7–29.9°
*c* = 13.0325 (3) Å	µ = 0.19 mm^−^^1^
α = 113.481 (1)°	*T* = 100 K
β = 90.942 (1)°	Block, yellow
γ = 107.359 (1)°	0.41 × 0.22 × 0.17 mm
*V* = 997.81 (4) Å^3^	

### Data collection

**Table:**

Bruker SMART APEXII CCD diffractometer	4542 independent reflections
Radiation source: fine-focus sealed tube	3499 reflections with *I* > 2σ(*I*)
Graphite monochromator	*R*_int_ = 0.031
φ and ω scans	θ_max_ = 27.5°, θ_min_ = 1.7°
Absorption correction: multi-scan (*SADABS*; Bruker, 2009)	*h* = −9→10
*T*_min_ = 0.925, *T*_max_ = 0.967	*k* = −14→14
15860 measured reflections	*l* = −16→16

### Refinement

**Table:**

Refinement on *F*^2^	Primary atom site location: structure-invariant direct methods
Least-squares matrix: full	Secondary atom site location: difference Fourier map
*R*[*F*^2^ > 2σ(*F*^2^)] = 0.044	Hydrogen site location: inferred from neighbouring sites
*wR*(*F*^2^) = 0.124	H atoms treated by a mixture of independent and constrained refinement
*S* = 1.04	*w* = 1/[σ^2^(*F*_o_^2^) + (0.0598*P*)^2^ + 0.5141*P*] where *P* = (*F*_o_^2^ + 2*F*_c_^2^)/3
4542 reflections	(Δ/σ)_max_ < 0.001
292 parameters	Δρ_max_ = 0.33 e Å^−^^3^
0 restraints	Δρ_min_ = −0.33 e Å^−^^3^

### Special details

**Table:**

Experimental. The crystal was placed in the cold stream of an Oxford Cryosystems Cobra open-flow nitrogen cryostat (Cosier & Glazer, 1986) operating at 100.0 (1) K.
Geometry. All e.s.d.'s (except the e.s.d. in the dihedral angle between two l.s. planes) are estimated using the full covariance matrix. The cell e.s.d.'s are taken into account individually in the estimation of e.s.d.'s in distances, angles and torsion angles; correlations between e.s.d.'s in cell parameters are only used when they are defined by crystal symmetry. An approximate (isotropic) treatment of cell e.s.d.'s is used for estimating e.s.d.'s involving l.s. planes.
Refinement. Refinement of *F*^2^ against ALL reflections. The weighted *R*-factor *wR* and goodness of fit *S* are based on *F*^2^, conventional *R*-factors *R* are based on *F*, with *F* set to zero for negative *F*^2^. The threshold expression of *F*^2^ > σ(*F*^2^) is used only for calculating *R*-factors(gt) *etc*. and is not relevant to the choice of reflections for refinement. *R*-factors based on *F*^2^ are statistically about twice as large as those based on *F*, and *R*-factors based on ALL data will be even larger.

### Fractional atomic coordinates and isotropic or equivalent isotropic displacement parameters (Å^2^)

**Table:**

	*x*	*y*	*z*	*U*_iso_*/*U*_eq_	
S1	0.57687 (7)	0.72177 (5)	0.01468 (4)	0.02486 (14)	
O1	−0.19366 (17)	0.59419 (13)	0.21670 (11)	0.0213 (3)	
O1W	0.3788 (2)	1.05291 (17)	0.10581 (15)	0.0346 (4)	
N1	−0.3199 (2)	0.39469 (16)	0.25166 (13)	0.0191 (3)	
N2	−0.2933 (2)	0.27084 (16)	0.23124 (14)	0.0206 (3)	
N3	0.1454 (2)	0.55365 (17)	0.11750 (14)	0.0232 (4)	
N4	0.3012 (2)	0.57326 (18)	0.07115 (14)	0.0233 (4)	
N5	0.3021 (2)	0.79095 (19)	0.10655 (15)	0.0248 (4)	
C1	0.0100 (2)	0.7186 (2)	0.39444 (16)	0.0198 (4)	
H1A	−0.0239	0.6386	0.4094	0.024*	
C2	0.1399 (2)	0.84368 (19)	0.47281 (16)	0.0197 (4)	
C3	0.2230 (3)	0.8533 (2)	0.57417 (16)	0.0216 (4)	
H3A	0.1904	0.7752	0.5916	0.026*	
C4	0.3494 (3)	0.9740 (2)	0.64713 (17)	0.0245 (4)	
H4A	0.4040	0.9788	0.7147	0.029*	
C5	0.3998 (3)	1.0916 (2)	0.62329 (17)	0.0237 (4)	
H5A	0.4885	1.1748	0.6744	0.028*	
C6	0.3209 (3)	1.0856 (2)	0.52650 (17)	0.0226 (4)	
H6A	0.3550	1.1652	0.5111	0.027*	
C7	0.1886 (2)	0.96195 (19)	0.44872 (16)	0.0192 (4)	
C8	0.1020 (3)	0.9522 (2)	0.34795 (16)	0.0220 (4)	
H8A	0.1321	1.0312	0.3317	0.026*	
C9	−0.0238 (3)	0.8314 (2)	0.27380 (16)	0.0217 (4)	
H9A	−0.0822	0.8266	0.2074	0.026*	
C10	−0.0654 (2)	0.71408 (19)	0.29778 (16)	0.0193 (4)	
C11	−0.5279 (3)	0.5262 (2)	0.30213 (16)	0.0229 (4)	
H11A	−0.4700	0.5811	0.2646	0.027*	
C12	−0.6720 (3)	0.5495 (2)	0.35584 (17)	0.0248 (4)	
H12A	−0.7104	0.6229	0.3569	0.030*	
C13	−0.7608 (3)	0.4676 (2)	0.40784 (17)	0.0258 (4)	
H13A	−0.8606	0.4835	0.4431	0.031*	
C14	−0.7026 (3)	0.3621 (2)	0.40795 (17)	0.0252 (4)	
H14A	−0.7634	0.3053	0.4432	0.030*	
C15	−0.5566 (3)	0.3391 (2)	0.35700 (16)	0.0222 (4)	
H15A	−0.5162	0.2675	0.3582	0.027*	
C16	−0.4695 (2)	0.42118 (19)	0.30412 (16)	0.0190 (4)	
C17	−0.1488 (2)	0.2736 (2)	0.18121 (16)	0.0203 (4)	
C18	−0.0785 (2)	0.3980 (2)	0.16783 (16)	0.0205 (4)	
C19	−0.1928 (2)	0.47093 (19)	0.21273 (16)	0.0191 (4)	
C20	0.0800 (3)	0.4335 (2)	0.11819 (16)	0.0223 (4)	
H20A	0.1373	0.3665	0.0853	0.027*	
C21	0.3835 (3)	0.6963 (2)	0.06758 (15)	0.0196 (4)	
C22	−0.0798 (3)	0.1541 (2)	0.14558 (18)	0.0269 (4)	
H22A	−0.1585	0.0808	0.1632	0.040*	
H22B	0.0410	0.1853	0.1863	0.040*	
H22C	−0.0768	0.1180	0.0639	0.040*	
H1N4	0.353 (3)	0.504 (3)	0.044 (2)	0.039 (7)*	
H2N5	0.346 (4)	0.872 (3)	0.103 (2)	0.049 (8)*	
H1N5	0.206 (3)	0.770 (3)	0.137 (2)	0.039 (7)*	
H2W1	0.367 (4)	1.077 (3)	0.045 (3)	0.066 (10)*	
H1W1	0.478 (4)	1.114 (4)	0.152 (3)	0.071 (10)*	

### Atomic displacement parameters (Å^2^)

**Table:**

	*U*^11^	*U*^22^	*U*^33^	*U*^12^	*U*^13^	*U*^23^
S1	0.0260 (3)	0.0226 (3)	0.0313 (3)	0.0092 (2)	0.0110 (2)	0.0157 (2)
O1	0.0210 (7)	0.0147 (7)	0.0275 (7)	0.0045 (5)	0.0013 (5)	0.0095 (6)
O1W	0.0398 (9)	0.0229 (8)	0.0378 (9)	0.0026 (7)	−0.0007 (8)	0.0156 (7)
N1	0.0204 (8)	0.0158 (8)	0.0233 (8)	0.0078 (6)	0.0040 (6)	0.0092 (7)
N2	0.0230 (8)	0.0155 (8)	0.0258 (8)	0.0080 (7)	0.0042 (7)	0.0098 (7)
N3	0.0234 (8)	0.0221 (9)	0.0245 (8)	0.0069 (7)	0.0065 (7)	0.0106 (7)
N4	0.0250 (9)	0.0214 (9)	0.0271 (9)	0.0100 (7)	0.0104 (7)	0.0118 (7)
N5	0.0272 (9)	0.0199 (9)	0.0307 (9)	0.0092 (7)	0.0101 (8)	0.0129 (8)
C1	0.0203 (9)	0.0176 (9)	0.0258 (10)	0.0082 (8)	0.0084 (8)	0.0118 (8)
C2	0.0197 (9)	0.0185 (9)	0.0243 (10)	0.0097 (8)	0.0081 (7)	0.0098 (8)
C3	0.0245 (10)	0.0190 (10)	0.0261 (10)	0.0102 (8)	0.0071 (8)	0.0119 (8)
C4	0.0255 (10)	0.0247 (11)	0.0240 (10)	0.0104 (8)	0.0040 (8)	0.0097 (9)
C5	0.0229 (10)	0.0193 (10)	0.0267 (10)	0.0064 (8)	0.0040 (8)	0.0079 (8)
C6	0.0243 (10)	0.0165 (9)	0.0295 (10)	0.0085 (8)	0.0091 (8)	0.0108 (8)
C7	0.0201 (9)	0.0183 (9)	0.0227 (9)	0.0097 (7)	0.0087 (7)	0.0095 (8)
C8	0.0261 (10)	0.0181 (9)	0.0269 (10)	0.0099 (8)	0.0091 (8)	0.0124 (8)
C9	0.0243 (10)	0.0208 (10)	0.0235 (10)	0.0091 (8)	0.0064 (8)	0.0117 (8)
C10	0.0157 (9)	0.0167 (9)	0.0255 (10)	0.0058 (7)	0.0061 (7)	0.0084 (8)
C11	0.0243 (10)	0.0219 (10)	0.0253 (10)	0.0095 (8)	0.0052 (8)	0.0115 (8)
C12	0.0256 (10)	0.0225 (10)	0.0302 (11)	0.0125 (8)	0.0046 (8)	0.0116 (9)
C13	0.0230 (10)	0.0276 (11)	0.0288 (11)	0.0111 (9)	0.0083 (8)	0.0119 (9)
C14	0.0247 (10)	0.0219 (10)	0.0292 (11)	0.0060 (8)	0.0073 (8)	0.0122 (9)
C15	0.0234 (10)	0.0184 (10)	0.0257 (10)	0.0076 (8)	0.0033 (8)	0.0098 (8)
C16	0.0177 (9)	0.0178 (9)	0.0202 (9)	0.0064 (7)	0.0029 (7)	0.0063 (8)
C17	0.0210 (9)	0.0167 (9)	0.0212 (9)	0.0055 (7)	0.0012 (7)	0.0068 (8)
C18	0.0218 (9)	0.0177 (9)	0.0226 (9)	0.0061 (8)	0.0037 (7)	0.0094 (8)
C19	0.0190 (9)	0.0152 (9)	0.0220 (9)	0.0033 (7)	0.0014 (7)	0.0086 (8)
C20	0.0237 (10)	0.0199 (10)	0.0257 (10)	0.0091 (8)	0.0053 (8)	0.0106 (8)
C21	0.0251 (10)	0.0169 (9)	0.0168 (9)	0.0065 (8)	0.0016 (7)	0.0077 (8)
C22	0.0270 (10)	0.0210 (10)	0.0348 (11)	0.0108 (8)	0.0083 (9)	0.0116 (9)

### Geometric parameters (Å, º)

**Table:**

S1—C21	1.6859 (19)	C6—C7	1.421 (3)
O1—C19	1.357 (2)	C6—H6A	0.9500
O1—C10	1.403 (2)	C7—C8	1.422 (3)
O1W—H2W1	0.94 (3)	C8—C9	1.369 (3)
O1W—H1W1	0.88 (3)	C8—H8A	0.9500
N1—C19	1.359 (2)	C9—C10	1.410 (3)
N1—N2	1.380 (2)	C9—H9A	0.9500
N1—C16	1.428 (2)	C11—C12	1.388 (3)
N2—C17	1.327 (2)	C11—C16	1.391 (3)
N3—C20	1.289 (2)	C11—H11A	0.9500
N3—N4	1.383 (2)	C12—C13	1.384 (3)
N4—C21	1.352 (2)	C12—H12A	0.9500
N4—H1N4	0.93 (3)	C13—C14	1.386 (3)
N5—C21	1.335 (3)	C13—H13A	0.9500
N5—H2N5	0.89 (3)	C14—C15	1.385 (3)
N5—H1N5	0.88 (3)	C14—H14A	0.9500
C1—C10	1.362 (3)	C15—C16	1.390 (3)
C1—C2	1.423 (3)	C15—H15A	0.9500
C1—H1A	0.9500	C17—C18	1.414 (3)
C2—C3	1.417 (3)	C17—C22	1.497 (3)
C2—C7	1.420 (3)	C18—C19	1.381 (3)
C3—C4	1.366 (3)	C18—C20	1.446 (3)
C3—H3A	0.9500	C20—H20A	0.9500
C4—C5	1.411 (3)	C22—H22A	0.9800
C4—H4A	0.9500	C22—H22B	0.9800
C5—C6	1.368 (3)	C22—H22C	0.9800
C5—H5A	0.9500		
			
C19—O1—C10	116.98 (14)	C1—C10—C9	122.20 (18)
H2W1—O1W—H1W1	107 (3)	O1—C10—C9	115.16 (16)
C19—N1—N2	110.16 (15)	C12—C11—C16	118.91 (18)
C19—N1—C16	131.06 (16)	C12—C11—H11A	120.5
N2—N1—C16	118.75 (15)	C16—C11—H11A	120.5
C17—N2—N1	105.70 (15)	C13—C12—C11	121.09 (19)
C20—N3—N4	114.56 (16)	C13—C12—H12A	119.5
C21—N4—N3	119.81 (16)	C11—C12—H12A	119.5
C21—N4—H1N4	118.9 (16)	C12—C13—C14	119.39 (18)
N3—N4—H1N4	121.3 (16)	C12—C13—H13A	120.3
C21—N5—H2N5	120.8 (18)	C14—C13—H13A	120.3
C21—N5—H1N5	117.0 (17)	C15—C14—C13	120.44 (19)
H2N5—N5—H1N5	122 (2)	C15—C14—H14A	119.8
C10—C1—C2	119.59 (17)	C13—C14—H14A	119.8
C10—C1—H1A	120.2	C14—C15—C16	119.67 (18)
C2—C1—H1A	120.2	C14—C15—H15A	120.2
C3—C2—C7	119.00 (17)	C16—C15—H15A	120.2
C3—C2—C1	121.73 (17)	C15—C16—C11	120.46 (17)
C7—C2—C1	119.27 (17)	C15—C16—N1	118.54 (17)
C4—C3—C2	120.58 (18)	C11—C16—N1	121.00 (17)
C4—C3—H3A	119.7	N2—C17—C18	111.53 (16)
C2—C3—H3A	119.7	N2—C17—C22	120.73 (17)
C3—C4—C5	120.78 (19)	C18—C17—C22	127.74 (17)
C3—C4—H4A	119.6	C19—C18—C17	104.38 (16)
C5—C4—H4A	119.6	C19—C18—C20	130.63 (18)
C6—C5—C4	119.91 (19)	C17—C18—C20	124.98 (17)
C6—C5—H5A	120.0	O1—C19—N1	122.18 (16)
C4—C5—H5A	120.0	O1—C19—C18	129.54 (17)
C5—C6—C7	120.90 (18)	N1—C19—C18	108.22 (16)
C5—C6—H6A	119.6	N3—C20—C18	121.63 (18)
C7—C6—H6A	119.6	N3—C20—H20A	119.2
C2—C7—C6	118.82 (17)	C18—C20—H20A	119.2
C2—C7—C8	118.63 (17)	N5—C21—N4	116.22 (17)
C6—C7—C8	122.55 (17)	N5—C21—S1	123.99 (15)
C9—C8—C7	121.42 (18)	N4—C21—S1	119.79 (14)
C9—C8—H8A	119.3	C17—C22—H22A	109.5
C7—C8—H8A	119.3	C17—C22—H22B	109.5
C8—C9—C10	118.83 (18)	H22A—C22—H22B	109.5
C8—C9—H9A	120.6	C17—C22—H22C	109.5
C10—C9—H9A	120.6	H22A—C22—H22C	109.5
C1—C10—O1	122.62 (17)	H22B—C22—H22C	109.5
			
C19—N1—N2—C17	0.7 (2)	C14—C15—C16—C11	0.0 (3)
C16—N1—N2—C17	179.09 (16)	C14—C15—C16—N1	−179.64 (17)
C20—N3—N4—C21	−178.93 (17)	C12—C11—C16—C15	1.4 (3)
C10—C1—C2—C3	−179.63 (17)	C12—C11—C16—N1	−178.98 (17)
C10—C1—C2—C7	0.0 (3)	C19—N1—C16—C15	−166.70 (19)
C7—C2—C3—C4	−0.7 (3)	N2—N1—C16—C15	15.3 (2)
C1—C2—C3—C4	178.93 (18)	C19—N1—C16—C11	13.6 (3)
C2—C3—C4—C5	0.1 (3)	N2—N1—C16—C11	−164.33 (17)
C3—C4—C5—C6	0.4 (3)	N1—N2—C17—C18	−0.1 (2)
C4—C5—C6—C7	−0.3 (3)	N1—N2—C17—C22	−179.62 (17)
C3—C2—C7—C6	0.8 (3)	N2—C17—C18—C19	−0.6 (2)
C1—C2—C7—C6	−178.83 (16)	C22—C17—C18—C19	178.93 (19)
C3—C2—C7—C8	−178.74 (16)	N2—C17—C18—C20	178.86 (17)
C1—C2—C7—C8	1.6 (3)	C22—C17—C18—C20	−1.6 (3)
C5—C6—C7—C2	−0.3 (3)	C10—O1—C19—N1	106.6 (2)
C5—C6—C7—C8	179.23 (18)	C10—O1—C19—C18	−76.5 (2)
C2—C7—C8—C9	−1.1 (3)	N2—N1—C19—O1	176.39 (16)
C6—C7—C8—C9	179.36 (18)	C16—N1—C19—O1	−1.7 (3)
C7—C8—C9—C10	−1.0 (3)	N2—N1—C19—C18	−1.1 (2)
C2—C1—C10—O1	179.76 (16)	C16—N1—C19—C18	−179.22 (18)
C2—C1—C10—C9	−2.2 (3)	C17—C18—C19—O1	−176.24 (18)
C19—O1—C10—C1	−24.8 (2)	C20—C18—C19—O1	4.3 (3)
C19—O1—C10—C9	157.06 (16)	C17—C18—C19—N1	1.0 (2)
C8—C9—C10—C1	2.8 (3)	C20—C18—C19—N1	−178.40 (19)
C8—C9—C10—O1	−179.09 (16)	N4—N3—C20—C18	177.44 (17)
C16—C11—C12—C13	−2.0 (3)	C19—C18—C20—N3	5.4 (3)
C11—C12—C13—C14	1.2 (3)	C17—C18—C20—N3	−173.91 (19)
C12—C13—C14—C15	0.3 (3)	N3—N4—C21—N5	−2.4 (3)
C13—C14—C15—C16	−0.9 (3)	N3—N4—C21—S1	178.15 (13)

### Hydrogen-bond geometry (Å, º)

**Table:**

*D*—H···*A*	*D*—H	H···*A*	*D*···*A*	*D*—H···*A*
N4—H1*N*4···S1^i^	0.93 (3)	2.56 (3)	3.466 (2)	165 (3)
N5—H2*N*5···O1*W*	0.89 (3)	1.94 (3)	2.805 (3)	164 (3)
O1*W*—H2*W*1···S1^ii^	0.94 (4)	2.58 (4)	3.397 (2)	145 (3)
O1*W*—H1*W*1···N2^iii^	0.89 (4)	2.01 (4)	2.876 (3)	167 (4)
C20—H20*A*···S1^i^	0.95	2.87	3.741 (2)	153

Symmetry codes: (i) −*x*+1, −*y*+1, −*z*; (ii) −*x*+1, −*y*+2, −*z*; (iii) *x*+1, *y*+1, *z*.
